# Community-associated Methicillin-resistant *Staphylococcus aureus*, Minnesota, 2000–2003

**DOI:** 10.3201/eid1110.050141

**Published:** 2005-10

**Authors:** Jessica M. Buck, Kathryn Como-Sabetti, Kathleen H. Harriman, Richard N. Danila, David J. Boxrud, Anita Glennen, Ruth Lynfield

**Affiliations:** * Minnesota Department of Health, Minneapolis, Minnesota, USA

**Keywords:** Staphylococcal infections, methicillin resistance, community-acquired infections, research

## Abstract

We compared characteristics of community-associated methicillin-resistant *Staphylococcus aureus* (CA-MRSA) skin and soft tissue infections (SSTIs) and CA-MRSA invasive disease identified in Minnesota from 2000 through 2003. A total of 586 patients with SSTIs and 65 patients with invasive disease were identified. Patients with invasive disease were more likely to be smokers (p = 0.03), and report a history of immunosuppressive therapy (p = 0.03), emphysema (p = 0.011), or injection drug use (p = 0.020) than were SSTI patients. Invasive disease isolates were less likely to be susceptible to ciprofloxacin (p = 0.002) and clindamycin (p = 0.001) and more likely to have healthcare-associated pulsed-field gel electrophoresis subtypes than SSTI isolates (p<0.001). Patients with invasive disease may have had healthcare exposures that put them at risk of acquiring healthcare-associated MRSA and which were not exclusion criteria in the CA-MRSA case definition. Continued surveillance of MRSA is needed to better characterize CA-MRSA infections.

## Abstract 2

Community-associated methicillin-resistant *Staphylococcus aureus* (MRSA) invasive disease resembled healthcare-associated MRSA disease.

Methicillin-resistant *Staphylococcus aureus* (MRSA) was first reported in 1961 and was recognized as a nosocomial pathogen by the late 1960s (*1**,**2*). Known MRSA risk factors include recent surgery or hospitalization, residence in a long-term care facility, presence of a percutaneous device or indwelling catheter, or recent dialysis (*3*).

In the 1980s, MRSA infections were reported in persons who lacked traditional MRSA risk factors. These infections appeared to be acquired in the community and are now known as community-associated (CA) MRSA infections. These infections have been reported worldwide (*4**–**18*). Outbreaks have occurred in many settings and among different populations (*10**,**16**,**19**–**23*).

Previous studies have demonstrated significant differences between MRSA isolates from persons with healthcare exposures and persons without these exposures in both antimicrobial susceptibility results and pulsed-field gel electrophoresis (PFGE) subtypes (*5**,**7*). Studies have also demonstrated demographic differences between CA-MRSA cases and healthcare-associated (HA) MRSA cases regarding age, race, and income (*5**,**7*).

The most common clinical manifestations of CA-MRSA are skin and soft tissue infections (SSTIs) such as abscesses or cellulitis (*5**,**7**,**9**,**24*). Less commonly, CA-MRSA can cause severe diseases such as necrotizing pneumonia, osteomyelitis, and septicemia (*6**–**9*). Most CA-MRSA infections resolve, but deaths from invasive CA-MRSA disease have been reported (*8*).

Although invasive disease caused by CA-MRSA has been described in the literature, no research has been published that evaluates possible patient and isolate differences between CA-MRSA SSTIs and CA-MRSA invasive disease. A subanalysis of CA-MRSA invasive disease patients and SSTI patients and isolates was conducted by using data collected from CA-MRSA prospective sentinel surveillance in Minnesota from 2000 through 2003.

## Methods

### Facility Enrollment

In 2000, 12 sentinel hospitals in Minnesota (6 in the 7-county Minneapolis–St. Paul metropolitan area and 6 in greater Minnesota) began reporting all cases of MRSA isolated in their respective microbiology laboratories to the Minnesota Department of Health (MDH). Characteristics of these sentinel sites have been described elsewhere (*7*).

### Case Enrollment

Infection control practitioners from each hospital completed a case report form for patients with a positive MRSA culture obtained during 2000–2003. Patient medical records were reviewed to determine the type of infection, history of underlying illness (injection drug use, diabetes, malignancy, chronic heart or lung conditions, chronic skin conditions), or immunosuppressive therapy (defined as long-term systemic steroid use, excluding topical creams, steroids used only for short-course treatment, and inhaled steroids used for asthma) and any history of patient healthcare exposures as defined in the CA-MRSA case definition. The hospital laboratories submitted CA-MRSA isolates to MDH.

All patients with cultures obtained during 2000–2002 who met the CA-MRSA case definition based on medical record review were interviewed to confirm their classification (patient culture dates 2000–2002) and to assess possible CA-MRSA risk factors (patient culture dates 2001–2002). Patients identified at 4 of the 12 sentinel sites during 2003 who had no exclusionary healthcare exposures noted on medical record review were contacted to confirm CA-MRSA classification and conduct risk factor interviews. In addition, a random sample of 2003 patients from the remaining 8 sentinel sites were interviewed to confirm CA-MRSA classification. Informed consent was obtained from all patients before telephone interview.

US Census data from 2000 were used to provide median income by zip code (*25*) as a proxy for case household income. The University of Minnesota and MDH Institutional Review Boards reviewed and approved the study.

### CA-MRSA Case Definition

The Centers for Disease Control and Prevention (CDC) Active Bacterial Core Surveillance Program defined a CA-MRSA case as a patient with an MRSA infection and no history of the following: surgery, hospitalization, or residence in a long-term care facility within the year before infection, presence of a percutaneous device or indwelling catheter, dialysis within the previous year, hospitalization >48 h before MRSA culture, or previous MRSA infection or colonization.

Patients were classified as confirmed CA-MRSA case-patients if the medical record review and interview did not show any of the above healthcare risk factors. Patients were classified as probable CA-MRSA case-patients if the medical record review did not show any healthcare risk factors, but the interview was not completed (because of patient refusal, inability to locate, or language barriers).

### Subanalysis Inclusion

CA-MRSA patients identified from prospective sentinel surveillance with culture dates in 2000 and 2003 were included in this subanalysis if they had an SSTI (e.g., abscess, cellulitis, folliculitis, wound infection [nonsurgical]) or infection in a normally sterile site caused by CA-MRSA. CDC's Active Bacterial Core Surveillance Program definition of sterile site infections was used to define cases of invasive CA-MRSA disease. This definition defines a normally sterile site as a portion of the body in a healthy state in which no microorganisms are found and includes the following: blood, cerebrospinal fluid, pleural fluid, peritoneal fluid, pericardial fluid, bone, joint fluid, internal body site (lymph node, brain, heart, liver, spleen, vitreous fluid, kidney, pancreas, or ovary), or other normally sterile site. Although cases of necrotizing pneumonia caused by CA-MRSA have been reported (*26*), CA-MRSA specimens isolated only from sputum were not included in our subanalysis because sputum was not defined as a sterile site.

### Isolate Characterization

All MRSA isolates submitted to MDH were tested to confirm *Staphylococcus aureus* identification by using a tube coagulase test (*27*) (Difco Laboratories, Detroit, MI, USA). Testing for antimicrobial susceptibility was performed by using a broth microdilution panel (PML Microbiologicals, Wilsonville, OR, USA) containing the following 11 antimicrobial agents: ciprofloxacin, gentamicin, trimethoprim/sulfamethoxazole, clindamycin, tetracycline, erythromycin, rifampin, linezolid, mupirocin, vancomycin, and oxacillin. Clinical and Laboratory Standards Institute (CLSI, formerly National Committee for Clinical Laboratory Standards) breakpoints were used to determine levels of resistance for all antimicrobial agents except mupirocin, for which no CLSI breakpoints exist (*28*). A standard of <4 μg/mL was used as a breakpoint for susceptibility to mupirocin (*29*).

### Molecular Characterization

Molecular subtyping of MRSA isolates was performed by PFGE and digestion with the restriction endonuclease *Sma*I (*30*). Patterns were evaluated both visually and with BioNumerics software (Applied Maths, Kortrijk, Belgium) by using the dice coefficient. Indistinguishable patterns must visually appear identical, and the DNA patterns must differ by <1.5% with respect to molecular weight. MRSA isolates were considered part of a CA-MRSA pulsed-field type (PFT) if they were >80% similar to the USA300 or USA400 reference strains based on Dice coefficients. MRSA isolates were considered part of an HA-MRSA PFT if they were >80% similar to USA100, USA200, or USA500–800 reference strains (*30*).

### Statistical Analysis

The Yates continuity corrected chi-square test was used to test for trends with EpiInfo version 6.2 (CDC, Atlanta, GA, USA), and univariate analysis of the data was performed with EpiInfo 2000 (CDC). Multivariate logistic regression was used to evaluate the association of the type of MRSA infection (SSTI versus invasive disease) with microbiologic and molecular features of the MRSA isolates. Demographic characteristics associated with the type of infection in the univariate analysis were controlled for in the multivariate analysis model. An α<0.05 significance level was required for predictors to remain in the model. Multivariate analysis was accomplished by using SAS version 8.0 for Windows (SAS Institute, Cary, NC, USA).

## Results

A total of 738 CA-MRSA infections were identified from January 1, 2000, to December 31, 2003. SSTIs accounted for 79% (586/738) of all infections reported, and invasive disease accounted for 9% (65/738) of all CA-MRSA infections reported. The proportion of CA-MRSA infections that were invasive did not differ significantly over the study period. The most common site of invasive disease was the bloodstream (50%), followed by joint or bone (32%). Clinical information was available for 511 (87%) of 586 SSTI patients. The most common clinical conditions reported for SSTIs were abscesses (49%) and cellulitis (33%) (Table 1).

**Table 1:** Community-associated methicillin-resistant Staphylococcus aureus invasive infection sites and skin and soft tissue infection clinical manifestations

Site or manifestation	No. (%)
Invasive infection site (n = 65)	
Bloodstream infection	
Without focus	25 (38)
With skin focus	6 (9)
With respiratory focus	2 (3)
Pleural fluid	3 (5)
Peritoneal fluid	2 (3)
Joint/bone	21 (32)
Other*	6 (9)
Skin and soft tissue clinical manifestation (n = 511†)	
Abscess	251 (49)
Cellulitis	171 (33)
Folliculitis	28 (5)
Wound infection	27 (5)
Impetigo	11 (2)
Other‡	62 (12)

*Other invasive isolate sources included brain tissue (*1*), lymph nodes (*2*), pancreatic aspirate (*1*), kidney abscess aspirate (*1*), and internal tissue (*1*). †A total of 411 skin and soft tissue infection patients had clinical manifestations reported. Results include multiple clinical manifestations per patient. ‡Other skin clinical manifestations included psoriasis, mastitis, cystic acne, furuncles, carbuncles, insect/spider bites, and eczema.

### Case Demographics

Invasive disease patients were more likely to be male than SSTI patients (66% vs. 51%, odds ratio [OR] 1.89, 95% confidence interval [CI] 1.10–3.24). No difference in median age was found between the 2 groups. Race information was available for 54 (83%) of 65 invasive disease patients and 477 (81%) of 586 SSTI patients. No difference was shown between the 2 groups when race was analyzed in terms of white and nonwhite race categories (Table 2).

**Table 2:** Community-associated methicillin-resistant Staphylococcus aureus invasive disease and skin and soft tissue infection (SSTI) patient demographics*

Characteristic	Invasive disease patient (n = 65)	SSTI patient (n = 586)	OR (95% CI)
Median age, y	28	24	NS
Sex (male), no. (%)	43 (66)	296 (51)	1.89 (1.10–3.24)
Race, no. (%)			
White†	33 (51)	254 (43)	NS
Unknown	10 (15)	105 (18)	
Nonwhite†	21 (32)	223 (38)	NS
Black	14 (21)	125 (21)	
Native American	6 (9)	82 (14)	
Asian	1 (0.5)	4 (0.7)	
Other	1 (0.5)	12 (0.2)	
Median income (US)‡	$38,237	$38,237	NS
Patients hospitalized, no. (%)§	49 (75)	173(31)	6.89 (3.81–12.4)
	(n = 58)	(n = 515)	
Presence of underlying condition, no. (%)	31 (53)	183 (36)	2.08 (1.21–3.60)
Immunosuppressive therapy,¶ no. (%)	3 (5)	3 (0.6)	9.31 (1.83–47.2)
Solid organ malignancy, no. (%)	2 (4)	2 (0.4)	9.16 (1.27–66.3)
Diabetes, no. (%)	9 (16)	41 (8)	NS
Current smoker, no. (%)	12 (21)	55 (11)	2.18 (1.09–4.37)
Emphysema/COPD, no. (%)	3 (5)	2 (0.4)	13.9 (2.29–85.5)
Injection drug use, no. (%)	3 (5%)	5 (1)	5.56 (1.29–23.9)

*OR, odds ratio; CI, confidence interval; NS, not significant; COPD, chronic obstructive pulmonary disease. †Race was calculated by using the number of cases with known race as the denominator. ‡Based on the mean household income in the zip code of each case-patient (source: 2000 US Census). §Calculated by using the number of cases with known hospitalization status. Twenty-four SSTI patients had unknown hospitalization status. ¶Defined as long-term systemic steroid use, excluding topical creams, steroids used only for short-course treatment, and inhaled steroids used for asthma.

Patient hospitalization status was available for all of the invasive disease CA-MRSA patients and 562 (96%) of 586 SSTI patients. As expected, invasive disease patients were more likely to be hospitalized for their infection than were SSTI patients (OR 6.89, 95% CI 3.81–12.4). Results remained significant after controlling for age and sex (p<0.001). No differences were observed between median income of CA-MRSA invasive disease patients and SSTI patients (Table 2).

History of underlying medical conditions was obtained for 58 (89%) of 65 invasive disease patients and 515 (88%) of 586 SSTI patients. Invasive disease patients were more likely to report a history of underlying illness than were SSTI patients (OR 2.08, 95% CI 1.05–4.20). Invasive disease CA-MRSA patients were more likely to have a history of immunosuppressive therapy (OR 9.31, 95% CI 1.87–47.2), solid organ malignancy (OR 9.16, 95% CI 1.27–66.3), or emphysema/chronic obstructive pulmonary disease (COPD) (OR 13.9, 95% CI 2.29–85.5) than SSTI patients. Invasive disease CA-MRSA patients were also more likely to be current smokers (OR 2.18, 95% CI 1.09–4.67) or injection drug users (OR 5.56, 95% CI 1.29–23.9) than SSTI patients. History of underlying illness (p = 0.007), immunosuppressive therapy (p = 0.003), emphysema/COPD (p = 0.012), current smoking (p = 0.028), and injection drug use (p = 0.021) remained significant in a multivariate model that controlled for age and sex (Table 2).

### Isolate Characteristics

We received isolates from 60 (92%) of 65 invasive disease patients and 525 (90%) of 586 SSTI patients. Tests for antimicrobial drug susceptibility were completed on 57 (95%) of 60 invasive disease isolates and 517 (98%) of 525 SSTI isolates. All isolates were susceptible to linezolid and vancomycin. Compared with SSTI isolates, those from invasive infections were less likely to be susceptible to ciprofloxacin (OR 2.79, 95% CI 1.54–5.04) and clindamycin (OR 3.34, 95% CI 1.67–6.69). When ciprofloxacin and clindamycin susceptibilities were analyzed in a model that controlled for sex and age, the results remained significant (p = 0.002 and p = 0.001, respectively) (Table 3).

**Table 3:** Community-associated methicillin-resistant Staphylococcus aureus (CA-MRSA) invasive disease patient and skin and soft tissue infection (SSTI) patient isolate characteristics*

Antimicrobial agent	Invasive disease isolates (n = 57), no. (% susceptible)	SSTI isolates (n = 517), no. (% susceptible)	OR (95% CI)	p value†	p value‡
Oxacillin (methicillin)	0	0	NA		
Ciprofloxacin	37 (65)	433 (84)	3.34 (1.67–6.69)	0.002	0.23
Clindamycin	44 (77)	475 (92)	2.79 (1.54–5.04)	0.001	0.20
Erythromycin	21 (37)	201 (39)	1.09 (0.62–1.92)		
Gentamicin	56 (98)	509 (98)	1.14 (0.14–9.25)		
Linezolid	57 (100)	517 (100)	NA		
Mupirocin	56 (98)	508 (98)	1.14 (0.14–9.23)		
Rifampin	56 (98)	515 (99)	4.60 (0.41–51.5)		
Tetracyline	52 (91)	469 (91)	0.94 (0.36–2.46)		
Trimethoprim-sulfamethoxazole	56 (98)	514 (99)	3.06 (0.31–2.99)		
Vancomycin	57 (100)	517 (100)	NA		

*OR, odds ratio; CI, confidence interval; NA, not applicable. †Refers to the probablility that the percentage susceptible for invasive CA-MRSA isolates differed from SSTI CA-MRSA isolates after controlling for sex and age. ‡Refers to the probability that the percentage susceptible for invasive CA-MRSA isolates differed from SSTI CA-MRSA isolates after controlling for sex and pulsed-field type associated with healthcare-associated MRSA or CA-MRSA.

Initial antimicrobial therapy information was documented for 41 (63%) of 65 patients with invasive disease whose isolates were available and for 415 (71%) of 586 patients with SSTI whose isolates were available. For 27 (66%) of 41 invasive disease patients and 333 (80%) of 415 SSTI patients, the initial antimicrobial agent prescribed was of a class to which the organism was resistant. Invasive disease patients were more likely to be empirically treated with an antimicrobial drug to which their MRSA isolate was susceptible than were SSTI patients (OR 2.10, 95% CI 1.05–4.20). Results remained significant after controlling for age and sex (p = 0.015).

All available isolates received were characterized by PFGE. Fifty-three (88%) of 60 invasive disease isolates and 501 (95%) of 525 SSTI isolates had PFGE subtypes that could be categorized into PFTs that have been associated with HA-MRSA (USA100, USA200, USA500–800) or CA-MRSA (USA300 and USA400) (*30*). Compared with PFGE subtypes from SSTI isolates, PFGE subtypes from invasive disease isolates were more likely to be associated with HA-MRSA PFTs (OR 3.63, 95% CI 2.03–6.50). This result remained significant after controlling for age and sex (p<0.001) (Table 4). When invasive disease and SSTI case isolate susceptibility to ciprofloxacin and clindamycin were analyzed in a multivariate model that controlled for CA- or HA-MRSA PFT and sex, no difference in susceptibility patterns was found between the 2 groups (Table 3).

**Table 4:** Distribution of HA-MRSA PFTs among invasive disease and SSTI community-associated MRSA isolates*

HA-MRSA PFTs	Invasive disease isolates (n = 25), no. (%)	SSTI isolates (n = 99), no. (%)
USA100	17 (28)	43 (8)
USA200	3 (5)	4 (0.8)
USA500	5 (8)	40 (7.6)
USA600	0	1 (0.2)
USA700	0	1 (0.2)
USA800	0	10 (2)
Total†	25 (42)	99 (19)

*HA, healthcare-associated; MRSA, methicillin-resistant *Staphylococcus aureus*; PFTs, pulsed-field types; SSTI, skin and soft tissue infection. †Odds ratio 3.63, 95% confidence interval 2.03–6.50, p<0.001.

### Confirmed CA-MRSA Analysis

Three hundred two (52%) of 586 SSTI patients and 36 (55%) of 65 invasive disease patients were confirmed (through patient interview and medical record review, as opposed to medical record review alone) to meet the CA-MRSA case definition. Confirmed CA-MRSA patients and isolates underwent the previously described analysis regarding differences in underlying conditions and isolate antimicrobial susceptibility. Underlying condition information was available for 30 (83%) of 36 invasive disease patients and 273 (90%) of 302 SSTI patients. Confirmed invasive disease CA-MRSA patients were more likely than confirmed SSTI patients to report a history of underlying illness (OR 2.36, 95% CI 1.09–5.10), history of immunosuppressive therapy (OR 10.0, 95% CI 1.92–52.0), solid organ malignancy (OR 19.4, 95% CI 1.71–221), or to be a current smoker (OR 3.06, 95% CI 1.25–7.50). Hospitalization information was available for all invasive disease patients and 299 (99%) of 302 SSTI patients. Confirmed invasive disease patients were more likely to be hospitalized for their infection than were confirmed SSTI patients (OR 5.94, 95% CI 2.75–12.8). Isolates were available for 30 (83%) of 36 invasive disease patients and 265 (88%) of 302 SSTI patients. Confirmed CA-MRSA invasive disease isolates were less likely to be susceptible to ciprofloxacin (OR 5.02, 95% CI 2.11–12.0) and clindamycin (OR 5.75, 95% CI 2.58–12.8). Twenty-eight (93%) of 30 invasive disease isolates and 257 (85%) of 302 SSTI isolates could be categorized into PFTs that have been associated with HA-MRSA or CA-MRSA. Invasive disease isolates were more likely to have HA-MRSA PFTs than were SSTI isolates (OR 4.24, 95% CI 1.90–9.43). When invasive disease and SSTI isolate susceptibility to ciprofloxacin and clindamycin were analyzed in a multivariate model that controlled for CA- or HA-MRSA PFT and sex, invasive disease isolates were still more likely to be resistant to ciprofloxacin than were SSTI isolates (p = 0.04).

## Discussion

This report compares CA-MRSA invasive disease patients and their isolates with those of SSTI patients. Invasive disease patients were more likely to be male and more likely to have a history of underlying conditions (immunosuppressive therapy, emphysema/COPD, injection drug use, and smoking) than were SSTI patients. Invasive disease isolates were similar to HA-MRSA isolates in that they were resistant to additional antimicrobial drugs (clindamycin and ciprofloxacin) and were more likely to belong to a PFT usually associated with HA-MRSA (*7*). These similarities suggest that invasive CA-MRSA patients may have had healthcare exposures that put them at risk of acquiring HA-MRSA, even though they are classified as CA-MRSA by the current CDC case definition.

The results of the ciprofloxacin and clindamycin multivariate analysis, including PFT association with both confirmed and probable CA-MRSA or HA-MRSA, showed no difference in susceptibility patterns between invasive disease and SSTI isolates. This suggests that the initial differences in susceptibility were not due to more resistant CA-MRSA strains causing invasive disease, but rather that more of the invasive disease isolates classified as CA-MRSA were actually HA-MRSA strains, which are typically resistant to more antimicrobial agents. However, when this same analysis was conducted by using confirmed CA-MRSA cases only, invasive disease isolates were still more likely to be resistant to ciprofloxacin. More research is needed to determine whether invasive disease CA-MRSA isolates are more resistant to antimicrobial drugs than CA-MRSA isolates that cause SSTI.

Invasive disease patient characteristics identified in this analysis were similar to results from other studies, which found that CA invasive disease patients had underlying conditions such as diabetes, smoking, and cardiovascular disease (*31**,**32*). The underlying conditions identified in the *S. aureus* and MRSA patients in these studies do not disqualify them from meeting the current CDC CA-MRSA case definition, yet these conditions may have put them at risk of acquiring HA-MRSA.

One possible explanation for some of the results of this analysis could be the likelihood that invasive disease patients had more healthcare exposures than did SSTI patients. This hypothesis is supported by the fact that invasive disease patients reported serious underlying illnesses that would imply a history of extensive healthcare contacts. During these healthcare contacts, invasive disease patients may have been colonized by HA-MRSA strains. A recent study found that in 50% of patients nasally colonized with MRSA subsequent infection developed over the next 18 months (*33*). Although we were unable to determine the colonization status of our patients for this analysis, patients have been found colonized with MRSA for up to 40 months (*34*).

This study has several limitations. Although the hospital laboratories were selected to reflect state population demographics, the study was not population based. Therefore, generalizing the findings to entire state population is not possible. Also, some HA-MRSA patients may have been misclassified as CA-MRSA patients because of incomplete ascertainment of HA risk factors. However, since no major differences were found in results when analysis was restricted to confirmed CA-MRSA patients, misclassification bias as a result of incomplete ascertainment of HA risk factors that are exclusion criteria for the current CA-MRSA case definition is unlikely to be a large factor. In addition, the sample size, particularly of invasive disease cases, limited the ability to detect small statistical differences between the 2 groups. Finally, complete data on all cases were not available for all factors analyzed in this report. These missing data could have biased the results of this analysis.

Underlying conditions or healthcare exposures not currently included as exclusion criteria in the CA-MRSA case definition may put patients at risk of HA-MRSA colonization and infection. In addition, persons with underlying conditions may also be at greater risk of invasive disease caused by MRSA. Clinicians should be aware of possible serious MRSA infections in persons without previously recognized HA-MRSA risk factors. Continued surveillance of CA-MRSA is needed to further define the epidemiology of invasive disease and SSTI and to develop recommendations for the prevention and control of this emerging public health threat.
